# A Trp574–Leu substitution confers resistance to three classes of acetolactate synthase inhibitors in Australian brome grass ( *B* *romus diandrus*)

**DOI:** 10.1002/ps.70979

**Published:** 2026-05-31

**Authors:** Roberto Busi, Danica Goggin, Nick Eyres, Fabian Runge, Aimone Porri, Ken Flower

**Affiliations:** ^1^ Australian Herbicide Resistance Initiative, UWA School of Agriculture and Environment University of Western Australia Perth Western Australia Australia; ^2^ Department of Primary Industry and Regional Development Western Australia Perth Western Australia Australia; ^3^ Greenough Specialty Agricultural Greenough Western Australia Australia; ^4^ IDENTXX GmbH Stuttgart Germany; ^5^ BASF SE, Herbicides Early Biology ‐ Global Research & Development Agricultural Solutions Limburgerhof Germany

**Keywords:** ALS‐inhibiting herbicides, brome grass, target‐site mutation, weed resistance

## Abstract

**BACKGROUND:**

Brome grass (*Bromus diandrus*) is a prevalent weed in southern Australian cropping regions which causes significant economic damage in crops and pastures. Before harvest 2023, two populations (named 6‐24 and 7‐24) suspected of resistance to imidazolinone herbicides were collected from separate fields. Preliminary screening indicated that these populations were resistant to several different acetolactate synthase (ALS)‐inhibiting herbicides. Therefore, further characterisation of the level and mechanism of resistance was carried out.

**RESULTS:**

There was high survival of the resistant populations at the maximum tested rates of sulfometuron (600 g ha^−1^) and imazamox + imazapyr (74 + 34 g ha^−1^, 2× the label rate for use in imidazolinone‐tolerant crops). All examined individuals (15 from each population) carried the same point mutation causing a tryptophan‐to‐leucine substitution at position 574 in the ALS protein. Application of the cytochrome P450 inhibitor malathion prior to herbicide treatment did not affect survival rates.

**CONCLUSIONS:**

This is the first study reporting a Trp574–Leu mutation as the basis for cross‐resistance to ALS‐inhibiting herbicides (sulfonylureas, imidazolinones and triazolopyrimidines) in Australian populations of *B. diandrus*. Control of these populations will require strategic use of pre‐emergence herbicides and herbicide‐tolerant crops in combination with harvest weed seed control. © 2026 The Author(s). *Pest Management Science* published by John Wiley & Sons Ltd on behalf of Society of Chemical Industry.

## Graphical abstract

Target‐site based imidazolinone resistance in brome grass was found to be conferred by a mutation at position 574 in the *ALS* gene and to severely impact the efficacy of the herbicide Intervix®, a co‐formulation of imazamox and imazapyr.

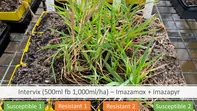

## 1 INTRODUCTION

The taxonomically complex genus *Bromus* contains 160–170 species of annual, biennial and perennial grasses, representing both agricultural weeds and forage crops.^1^ Many of the annual species grow in disturbed habitats, have weedy characteristics, and likely evolved in response to agricultural practices.^1^ A recent survey of Australian grain growers indicated that *Bromus* spp. (predominantly *B. diandrus* and *B. rigidus*) were ranked in their top five most problematic weeds and were perceived to be rapidly adapting to changes in both agricultural practices and climate.^2^ This is corroborated by economic modelling, which in 2016 identified *Bromus* spp. as an emerging problem in Australian agriculture,^3^ but in 2025 ranked *Bromus* second only to the major weed *Lolium rigidum*, costing A$23 million annually in yield revenue losses.^4^

Similar to *L. rigidum*, *Bromus* spp. have evolved significant levels of herbicide resistance in response to intensive chemical weed control efforts, a problem that is exacerbated by the increased adoption of minimum‐tillage cropping systems (highly reliant on herbicides) and the lack of selective herbicides which are effective on this genus.^5^ Unlike *L. rigidum*, however, *Bromus* weeds are polyploid (*B. rigidus* is hexaploid, *B. diandrus* octoploid),^6^ which has implications for the evolution and phenotypic expression of resistance traits. The relative rate of resistance evolution in polyploid species is likely to be a complex interaction between the dominance of the resistance trait, the ‘masking effect’ of multiple genomes on both beneficial and deleterious gene mutations, and the mating system (self‐ or cross‐pollinated) of the polyploid species.^7^ Worldwide, herbicide resistance has been reported in populations from ten different *Bromus* species, with seven species having populations resistant to the widely used acetolactate synthase (ALS)‐inhibiting herbicides.^8^ The target enzyme of these herbicides is part of the biosynthetic pathway for branched‐chain amino acids, so inhibition of ALS results in the slow depletion of amino acids and protein in the meristem, ultimately resulting in plant death.^9^ Most cases of resistance to the ALS inhibitors are due to point mutations at one of nine amino acid positions in the ALS enzyme (Ala122, Pro197, Ala205, Phe206, Asp376, Arg377, Trp574, Ser653 or Gly654),^10^ which decrease its binding affinity for the herbicides, but cases of enhanced metabolic detoxification of ALS inhibitors have also been reported.^9^

Target‐site mutations in ALS often result in very high resistance indices (>100), and cross‐resistance between two or more of the six chemical classes of ALS inhibitors, i.e. sulfonylureas (SU), imidazolinones (IMI), triazolopyrimidines (TP), triazolinones (SCT), sulfoanilides (SA) and pyrimidinyl benzoates (PTB), is common. In *B. tectorum* populations from the USA and *B. japonicus* populations from China, target‐site mutations at the Pro197, Asp376 or Ser653 amino acid positions resulted in cross‐resistance between the SU (e.g. sulfosulfuron, mesosulfuron), SCT (e.g. propoxycarbazone‐sodium), TP (e.g. pyroxsulam) and/or IMI (e.g. imazamox) classes of ALS inhibitors.^10^, ^11^, ^12^, ^13^, ^14^, ^15^ In a wide‐ranging survey of 49 sulfosulfuron‐resistant *B. tectorum* populations from Oregon, USA, mutations at Ala122, Ala205 or Trp574 were identified in addition to those listed above, with some populations carrying two or three different mutations (in different individuals).^16^ A target‐site mutation was suspected in a pyroxsulam‐resistant *B. diandrus* population from New Zealand, but the gene sequence was not determined,^17^ while a Trp574–Leu substitution in a *B. commutatus* population from the UK conferred cross‐resistance to SUs and pyroxsulam.^18^ In a different mode of target‐site resistance, a *B. sterilis* population from the Czech Republic was shown to have higher levels of *ALS* expression, so that adequate enzyme activity could be retained in the presence of the herbicide.^19^

There is also evidence that some ALS inhibitor‐resistant *Bromus* populations have enhanced rates of herbicide detoxification mediated by cytochrome P450 monooxygenases (P450s). Based on the ability of applied P450 inhibitors to decrease or reverse resistance, mesosulfuron‐resistant *B. japonicus* from China,^20^ imazamox‐resistant *B. tectorum* from Kansas,^11^ SU‐, TP‐ and SCT‐resistant *B. sterilis* from the Czech Republic,^19^ and SU‐resistant *B. rigidus* from Australia^21^ were deduced to possess an enhanced ability to detoxify the relevant herbicides. A SU‐ and IMI‐resistant *B. tectorum* from Oregon that lacked target‐site ALS mutations was directly shown to metabolise propoxycarbazone‐sodium at higher rates than susceptible and target‐site‐resistant populations in a reaction suppressed by a P450 inhibitor,^22^ and an investigation of candidate genes implicated a glutathione transferase isoform and an ATP‐binding cassette transporter in non‐target‐site‐based resistance to ALS inhibitors in *B. commutatus* from the UK^18^ and *B. japonicus* from China,^23^ respectively.

*Bromus diandrus* populations randomly collected from the Western Australian grain belt in late 2010 were all identified as being susceptible to SU and IMI‐type ALS‐inhibiting herbicides, whereas 13% of surveyed *B. rigidus* populations were classed as resistant.^24^ Since that time, however, *B. diandrus* populations surviving the recommended field rates of SUs and IMIs have been identified during targeted resistance tests.^25^ The aim of the current study was to characterise the level and mechanism of ALS inhibitor resistance in two of these populations.

## 2 MATERIALS AND METHODS

### 2.1 Plant material

*Bromus diandrus* populations with suspected resistance to IMI herbicides were collected from the Western Australian grain belt during the 2021, 2022, 2023 and 2024 harvests, as part of the University of Western Australia's herbicide resistance testing service.^25^ Populations 178‐21 (<6% plant survival at the recommended IMI field rate), 6‐24 and 7‐24 (both with 100% survival; see Supporting Information, Fig. S1 for their collection site) were singled out for more detailed study on their response to the different chemical classes of ALS inhibitors. A population with 0% survival to all herbicides tested, designated 1‐23, was used as a susceptible control.

### 2.2 Herbicide treatments

*Bromus diandrus* seeds were sown into commercial potting mix (50% river sand, 25% peat moss, 25% composted pine bark) in 12‐cell seedling trays and maintained in a naturally lit glasshouse at temperatures of 18–22 °C during the growing season (May–August) of each year. Cells were kept moist by watering to 80% field capacity, and when seedlings had reached the two‐leaf stage, they were counted, treated with herbicide and then returned to the glasshouse for another 28 days. All herbicides were applied post‐emergence using a custom‐built cabinet sprayer equipped with twin TeeJet XR11001 flat‐fan nozzles (Spraying Systems Co., Wheaton, IL, USA), calibrated to deliver 107 L of spray solution per ha at 210 kPa and a boom speed of 3.6 m s^−1^.^26^ Treated plants were returned to the glasshouse, and at 28 days after treatment, plants showing healthy new growth from the basal meristem were counted as survivors (Supporting Information, Fig. S2). Plant survival was expressed as a percentage of the total number of seedlings treated with herbicide.

#### 2.2.1 Preliminary screening

Preliminary resistance screening was performed on one replicate of 25 seedlings for each population, using the recommended field rates of the ALS‐inhibiting herbicides sulfometuron, sulfosulfuron, pyroxsulam and imazamox + imazapyr as detailed in Supporting Information, Table S1.

#### 2.2.2 Dose–response assays

Following identification of populations with putative resistance to ALS inhibitors in the preliminary screening, two independent dose–response experiments (see Table 1 for the list of treatments) were performed with each of sulfometuron and imazamox + imazapyr on populations 1‐23, 178‐21, 6‐24 and 7‐24. These two herbicides were selected for the dose–response study because they are the most commonly used ALS inhibitors for brome grass control in Australia. The high survival of populations 6‐24 and 7‐24 in the first experiment, in which the maximum doses used were 16× and 12× the recommended rate for sulfometuron and imazamox + imazapyr, respectively, prompted the use of higher maximum rates (32× and 64× the recommended rate) in the second experiment (Table 1). There were eight replicates of ten seedlings for each population and treatment.

**Table 1:** Herbicide treatments applied to *Bromus diandrus* populations for the dose–response study. The recommended field rate is shown in bold

Active ingredient	Formulation	Adjuvant	Dose (g ha^−1^)
Sulfometuron	Oust (750 g kg^−1^ sulfometuron‐methyl)^a^	None	Expt 1: 0, 3, 6, 12, **25**, 50, 100, 200, 300, 400 Expt 2: 0, 6, 12, **25**, 50, 100, 200, 400, 800
Imazamox + imazapyr	Intervix (33 g L^−1^ imazamox + 15 g L^−1^ imazapyr)^b^	1% Hasten^c^	Expt 1: 0, 0.6 + 0.3, 2.1 + 0.9, 4.1 + 1.9, 8.3 + 3.8, **16.5 + 7.5**, 33 + 15, 49.5 + 22.5, 66 + 30, 99 + 45, 132 + 60, 165 + 75, 198 + 90 Expt 2: 0, 2.1 + 0.9, 4.1 + 1.9, 8.3 + 3.8, **16.5 + 7.5**, 24.8 + 11.3, 33 + 15, 66 + 30, 132 + 60, 264 + 120, 528 + 240, 1056 + 480

^a^ Dupont Australia, Sydney, Australia.

^b^ BASF Australia Ltd, Melbourne, Australia.

^c^ Vicchem, Melbourne, Australia.

#### 2.2.3 Effect of malathion on the response to ALS inhibitors

The same populations as used in the dose–response study were also treated with single doses of the recommended field rate of sulfometuron, sulfosulfuron, pyroxsulam or imazamox + imazapyr in the presence and absence of the cytochrome P450 inhibitor malathion, which was applied 30 min prior to herbicide treatment, at a rate of 1000 g ha^−1^ (Table 2). There were three replicates of ten seedlings for each population and treatment. Pilot studies showed that malathion applied alone had no effect on plant growth or survival (data not shown).

**Table 2:** Treatments applied to *Bromus diandrus* populations to assess the effect of malathion on plant response to acetolactate synthase‐inhibiting herbicides. All herbicides were applied in the presence and absence of a malathion pre‐treatment

Active ingredient	Formulation	Adjuvant	Dose (g ha^−1^)
Sulfometuron	Oust (750 g kg^−1^ sulfometuron‐methyl)^a^	None	25
Sulfosulfuron	Monza (750 g kg^−1^ sulfosulfuron)^b^	None	50
Pyroxsulam	Crusader (30 g L^−1^ pyroxsulam + 90 g L^−1^ cloquintocet‐mexyl)^c^	1% BS1000^b^	140
Imazamox + imazapyr	Intervix (33 g L^−1^ imazamox + 15 g L^−1^ imazapyr)^d^	1% Hasten^e^	16.5 + 7.5
Malathion	David Gray's Malathion Garden Spray (500 g L^−1^ malathion)^f^	None	1000

^a^ Dupont Australia, Sydney, Australia.

^b^ Nufarm Australia, Laverton North, Australia.

^c^ Dow AgroSciences Australia Ltd, Sydney, Australia.

^d^ BASF Australia Ltd, Melbourne, Australia.

^e^ Vicchem, Melbourne, Australia.

^f^ David Gray & Co Pty Ltd, Perth, Australia.

### 2.3 ALS gene sequencing

Single leaves from untreated plants from susceptible population 1‐23, or the low SU‐resistant population 178‐21, were bulked from five individuals. Single leaves from individuals of resistant populations 6‐24 and 7‐24 were collected as independent individual samples, with five replicates each sampled from untreated plants, survivors from the sulfometuron dose–response study, and survivors from the imazamox + imazapyr dose–response study (15 individuals per population were sampled in total). Leaf samples were air‐dried and sent to IDENTXX GmbH (Stuttgart, Germany: www.identxx.com) for DNA extraction and sequencing of the *ALS* gene at the commonly mutated codon positions of Pro197, Trp574 and Ser653 (codon positions are relative to the *Arabidopsis thaliana* ALS protein sequence).

DNA was extracted, amplified, and sequenced by IDENTXX GmbH using the standard procedures described in detail in Busi *et al*.^25^ Polymerase chain reaction (PCR) amplification with proprietary primers for positions 197, 574 and 653 in the *ALS* gene was performed using a MangoTaq DNA polymerase (Bioline, Luckenwalde, Germany) and standard amplification conditions.^25^ PCR products were checked by agarose gel electrophoresis and analysed for single nucleotide polymorphisms via pyrosequencing using specific pyrosequencing primers (IDENTXX GmbH).^25^ Randomly selected samples were also sequenced using the Sanger method (SeqLab‐Microsynth, Göttingen, Germany) to validate the results of the pyrosequencing. Sanger chromatograms were analysed using Geneious software (v.9.1.8; Biomatters, Auckland, New Zealand).

### 2.4 Data analysis

Dose–response data were fitted to a three‐parameter non‐linear logistic model using the drc package^27^ in the statistical software R^28^ (v.4.3.0):

$$y=\frac{d}{1+\exp\left[b\left(\log\;x-\log e\right)\right]}$$

where *y* is per cent plant survival, *d* is the upper asymptotic value of *y* (100%), *b* is the slope of the curve, *e* is the LD_50_, that is the herbicide dose required to kill 50% of the population, and *x* is the herbicide dose (g ha^−1^). The *t*‐test functionality in the drc package was used to identify significant differences between LD_50_ values.

Differences in plant survival of herbicide treatments in the presence or absence of malathion were assessed with the max‐*t*‐test using the multcomp^29^ and sandwich^30^ packages in R as described by Herberich *et al*.^31^

## 3 RESULTS

### 3.1 Response of *Bromus diandrus* populations to ALS‐inhibiting herbicides

**Figure 1:**
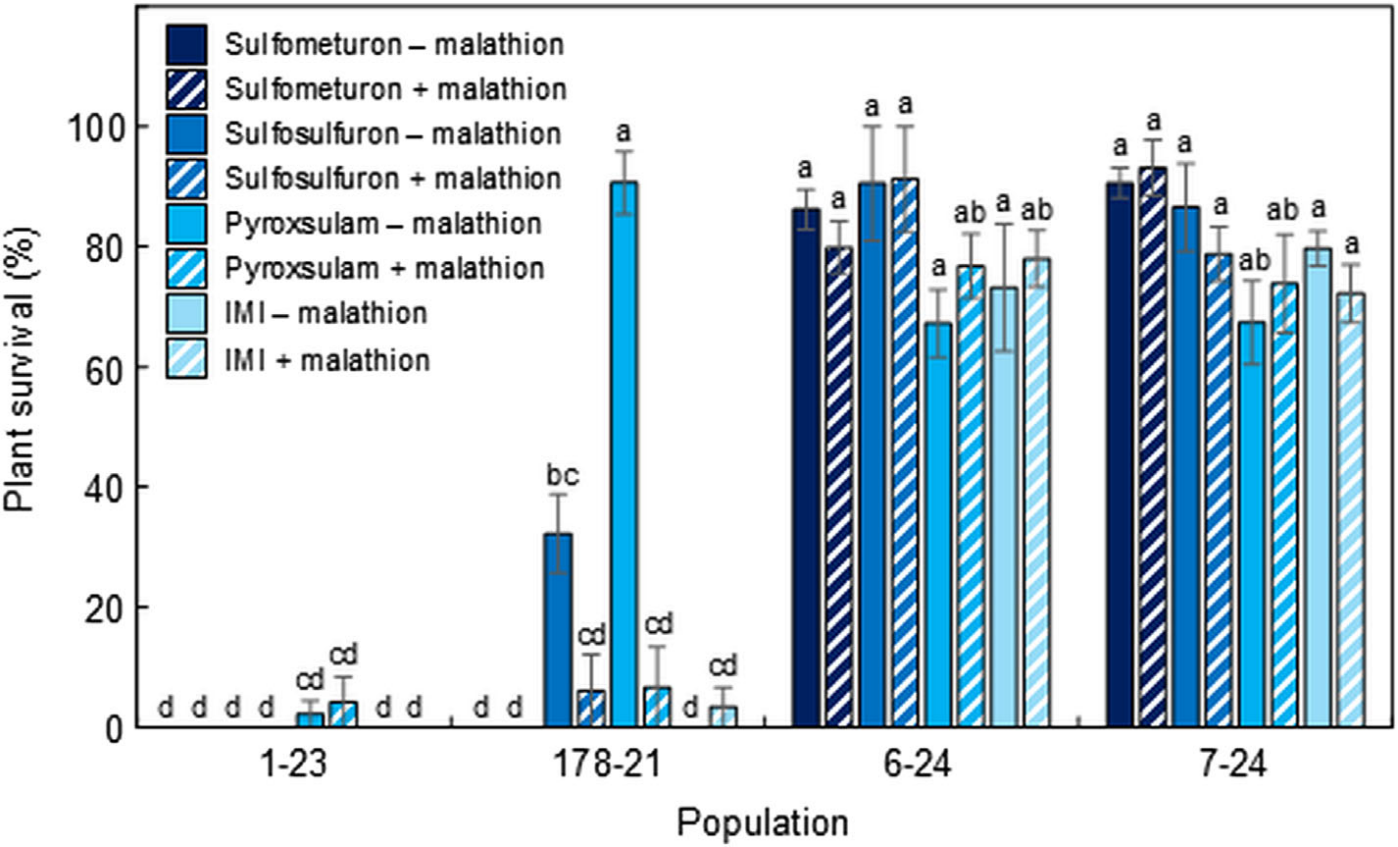
Response of populations to acetolactate synthase (ALS)‐inhibiting herbicides applied in the presence or absence of the cytochrome P450 inhibitor malathion. Values are means ± SE (*n* = 3); different letters above bars denote significant (*P* < 0.05) differences among populations and treatments. IMI, imazamox + imazapyr.

In the preliminary screening, susceptible population 1‐23 was fully controlled by the recommended rates of sulfosulfuron, sulfometuron, pyroxsulam and imazamox + imazapyr (Supporting Information, Table S2). Population 178‐21 was also fully controlled by sulfometuron and imazamox + imazapyr, but ~90% of individuals survived pyroxsulam and sulfosulfuron (Supporting Information, Table S2). All tested individuals from populations 6‐24 and 7‐24 survived the recommended rates of sulfometuron, sulfosulfuron, imazamox + imazapyr and pyroxsulam (Supporting Information, Table S2). Malathion had no significant effect on the (high‐level) survival of populations 6‐24 and 7‐24 to any of the ALS‐inhibiting herbicides tested, but caused a significant decrease in the survival of pyroxsulam‐treated population 178‐21 (Fig. 1). Population 1‐23 showed little to no survival to all herbicides tested, regardless of whether malathion was present (Fig. 1).

Quantification of resistance levels to sulfometuron and imazamox + imazapyr using a dose–response analysis revealed that the LD_50_ values of population 178‐21 were not significantly different from those of susceptible population 1‐23 for both sulfometuron and imazamox + imazapyr, and thus this population cannot be classed as resistant to these herbicides (Table 3). By contrast, sulfometuron and imazamox + imazapyr were largely ineffective on populations 6‐24 and 7‐24 (Supporting Information, Fig. S3), so that the resistance index was >116 for sulfometuron and imazamox + imazapyr (Table 3).

**Table 3:** Quantification of resistance levels to sulfometuron and imazamox + imazapyr. Plant survival data at various rates of each herbicide were fitted to dose–response curves to determine the dose at which 50% of the population was killed (LD_50_ ± standard error), and the resistance index (RI ± standard error), calculated by dividing the LD_50_ value of the (putative) resistant population by that of susceptible population 1‐23. An exact LD_50_ for populations 6‐24 and 7‐24 treated with sulfometuron could not be calculated, as plant survival was 100% at all herbicide doses. For the imazamox + imazapyr co‐formulation, the LD_50_ represents the sum of the two active ingredients. *P* < 0.05 indicates a significant difference between resistant and susceptible populations.

Population	Sulfometuron			Imazamox + imazapyr		
	LD_50_ (g ha^−1^)	RI	*P* value	LD_50_ (g ha^−1^)	RI	*P* value
1‐23	6.9 ± 1.7	—	—	2.8 ± 0.1	—	—
178‐21	14.5 ± 1.1	2.1 ± 0.7	0.137	2.6 ± 0.7	0.9 ± 0.3	0.779
6‐24	>800	>116	nd^a^	757 ± 11	271 ± 11	<0.001
7‐24	>800	>116	nd	2887 ± 56	1040 ± 204	<0.001

^a^ nd, not determined

### 3.2 *ALS* gene sequencing

Pyrosequencing of the regions around the codons for Pro197, Ser653 and Trp574 (Table 4) revealed wild‐type sequences for all batches of plants from IMI‐susceptible populations 1‐23 and 178‐21. By contrast, a G‐to‐T point mutation in codon 574, causing a Trp‐to‐Leu amino acid substitution, was detected in all sequenced individuals from populations 6‐24 and 7‐24, while there were wild‐type codons at Pro197 and Ser653 (Table 4). The fact that signals for both Trp (wild type) and Leu (mutant) codons were detected at position 574 in each of the sequenced 6‐24 and 7‐24 individuals (Supporting Information, Fig. S4.) suggests that in octoploid *B. diandrus*, the resistance mutation is not present in all four genomes.

**Table 4:** Sequencing of the *ALS* gene at amino acid positions 197, 574 and 653 in imazamox + imazapyr (IMI) susceptible and resistant populations of *Bromus diandrus*. For the highly resistant populations 6‐24 and 7‐24, leaf samples were taken from individuals that had not received herbicide treatment, as well as from survivors of sulfometuron and imazamox + imazapyr treatments.

			Position 197		Position 574		Position 653	
Population	Treatment	No. of plants	Codon sequence	Amino acid	Codon sequence	Amino acid	Codon sequence	Amino acid
1‐23	None	5 (bulked)	CCC	Pro	TGG	Trp	AGC	Ser
6‐24	None	5 (individuals)^c^	CCC	Pro	T(G/T)G	Trp/Leu	AGC	Ser
6‐24	SU^a^	5 (individuals)^c^	CCC	Pro	T(G/T)G	Trp/Leu	AGC	Ser
6‐24	IMI^b^	5 (individuals)^c^	CCC	Pro	T(G/T)G	Trp/Leu	AGC	Ser
7‐24	None	5 (individuals)^c^	CCC	Pro	T(G/T)G	Trp/Leu	AGC	Ser
7‐24	SU^a^	5 (individuals)^c^	CCC	Pro	T(G/T)G	Trp/Leu	AGC	Ser
7‐24	IMI^b^	5 (individuals)^c^	CCC	Pro	T(G/T)G	Trp/Leu	AGC	Ser
178‐21	None	5 (bulked)	CCC	Pro	TGG	Trp	AGC	Ser

^a^ Plants were taken from the cohort surviving sulfometuron (19–600 g ha^−1^).

^b^ Plants were taken from the cohort surviving imazamox + imazapyr (24–768 g ha^−1^ total IMI).

^c^ Each sequenced individual gave the same result.

## 4 DISCUSSION

This study reports the first known case of target‐site‐based cross‐resistance to ALS‐inhibiting herbicides in Australian populations of *Bromus diandrus*, a weed with steadily increasing economic impact in Australian cropping systems. All tested individuals from two populations with high levels of survival to SU‐, TP‐ and IMI‐class ALS inhibitors (populations 6‐24 and 7‐24) had a tryptophan‐to‐leucine amino acid substitution at position 574 of the ALS protein, and the ineffectiveness of the cytochrome P450 inhibitor malathion in reversing resistance indicated that enhanced herbicide metabolism was unlikely to contribute greatly to resistance in these populations. Across all reported cases of evolved target‐site resistance to ALS inhibitors, the Trp574–Leu substitution is the second most common mutation after Pro197,^9^ and is known to confer broad cross‐resistance to ALS‐inhibiting herbicides.^32^

A previous study on a Western Australian *B. diandrus* population with target‐site resistance to acetyl‐CoA carboxylase (ACCase)‐inhibiting herbicides indicated that the mutation was homozygous in one of the genomes of this octoploid species, whereas the other three genomes had the susceptible genotype: this was sufficient to confer 5–44‐fold resistance depending on the chemical class of the ACCase inhibitor and the temperature at the time of application^25^ The Trp574–Leu ALS mutation detected in the populations characterised in the current study is also unlikely to be present in all genomes, as suggested by the heterozygous nature of the sequencing results (Table 3), but this was not investigated further. The fact that these populations were >100‐fold more resistant to SU and IMI herbicides than the susceptible control population indicates that full homozygosity of the Trp574–Leu mutation is not necessary for high‐level resistance. There is often a gene‐dilution effect on resistance mutations in polyploid species compared with diploids (reviewed by Kaundun^33^), but the situation is highly complex, because the potential fitness penalties of resistance mechanisms can also be diluted, allowing the population to persist and adapt more rapidly.^7^ In an elegant study on ALS mutations in hexaploid *Echinochloa crus‐galli*, Cutti *et al*.^34^ reported that a population with a Trp574–Leu mutation in ALS from subgenome C, the most‐expressed subgenome, was more resistant to ALS inhibitors (resistance index 35–31 000) than populations with the same mutation in ALS from subgenome A (the least‐expressed genome; resistance index 7–4200). Therefore, in the highly resistant *B. diandrus* populations 6‐24 and 7‐24, it is possible that the ALS mutation occurs on a dominant subgenome, but this needs to be confirmed.

A third *B. diandrus* population with suspected IMI resistance (178‐21) proved to have negligible levels of resistance to SU or IMI herbicides, and this was borne out by its lack of ALS mutations at positions Pro197, Trp574 or Ser653. However, there were indications from the malathion treatment that its ability to survive pyroxsulam (a TP‐class ALS inhibitor) could be due to enhanced metabolism of this herbicide. A *B. sterilis* population from the Czech Republic was also hypothesised to metabolise pyroxsulam based on its response to malathion, and was shown to be cross‐resistant to SU‐ and SCT‐class ALS inhibitors.^19^

Several studies in other genera of grass weeds (e.g. *Alopecurus myosuroides* and *A. aequalis*, *Apera spica‐venti* and *Beckmannia syzigachne*), have also shown that in populations with metabolic resistance to ALS inhibitors (either implied by increased herbicide sensitivity in the presence of malathion or demonstrated by direct measurement of herbicide metabolism), there is usually cross‐resistance between the chemical classes.^35^, ^36^, ^37^, ^38^ The major metabolite of pyroxsulam in both domesticated (wheat) and wild (*Setaria viridis*, *S. pumila*, *A. myosuroides*) grasses is 5‐hydroxypyroxsulam, resulting from *O*‐demethylation of the triazolopyrimidine ring in a reaction characteristic of cytochrome P450.^39^, ^40^ CYP709C56, a P450 isolated from mesosulfuron‐methyl‐resistant *A. aequalis*, also *O*‐demethylates this herbicide; and transgenic *Arabidopsis* plants overexpressing CYP709C56 were resistant to pyroxsulam as well as to mesosulfuron‐methyl.^36^ By contrast, two P450s (CYP99A44 and CYP704A177) identified as potentially mediating metabolic resistance to mesosulfuron‐methyl in *Beckmannia syzigachne* by virtue of their higher expression levels in resistant plants, respectively conferred resistance in transgenic *Arabidopsis* to mesosulfuron‐methyl but not pyroxsulam or imazethapyr, or to pyroxsulam but not mesosulfuron‐methyl or imazethapyr.^41^ These two studies highlight the fact that metabolic cross‐resistance can either be conferred by a single, broad‐specificity enzyme metabolising multiple herbicides, or by several enzymes with high substrate specificity. In light of these findings, it would be worth characterising pyroxsulam metabolism at the molecular and chemical level in *B. diandrus*, as well as investigating the levels of non‐target‐site‐based resistance to various ALS inhibitors that can be achieved under selection pressure with pyroxsulam. In some cases, metabolic resistance to unrelated modes of action can also arise from an acquired ability to metabolise a particular herbicide, even if a population has not yet been exposed to those modes of action.^42^

With *Bromus* spp. increasing in importance as weeds of southern Australian cropping systems, it is necessary to be proactive in controlling herbicide‐resistant populations before they become dominant in a field. Because *Bromus* spp. are self‐pollinating, the spread of resistance will occur mainly through seed dispersal.^5^ Although the major weed of Australian grain production, *L. rigidum*, has seeds that have difficulty in emerging from burial depths beyond 5 cm, *B. diandrus* seeds are capable of emerging from depths of 10–20 cm, meaning that soil inversion is unlikely to be as successful in controlling *B. diandrus* as it is for *L. rigidum*.^43^ Another problem from a seed bank perspective is that *B. diandrus* tends to germinate in two cohorts, with the later cohort emerging with the crop.^5^ This, along with the ability of *B. diandrus* to shorten its life cycle under warm and dry conditions,^2^ means that control of this weed with post‐emergence herbicides can become very difficult. Another important issue is the increasing selection pressure from more frequent use of IMI‐tolerant crops in the rotation. Effective control of *Bromus* spp. requires the use of several different techniques within the same season, over three to four years, to ensure depletion of the seed bank: for example, using knockdown herbicides in years where a large proportion of weed seedlings have emerged before crop planting; use of pre‐emergence herbicides when conditions are suitable; planting of break crops to increase the range of post‐emergence herbicide options; and late‐season control by chemical or physical methods, to prevent seed set.^44^ Good results have been achieved using the pre‐emergence herbicide pyroxasulfone to control *B. tectorum* populations in North America, as long as there is sufficient rainfall after herbicide application.^45^ Successful control of IMI‐resistant *B. diandrus* has been achieved with the choice of a robust pre‐emergence herbicide such as propyzamide or carbetamide in grain legumes; with the use of quizalofop applied post‐emergence either in legumes or in quizaolfop‐tolerant (Co‐Axium) barley varieties; or by brown‐manuring with glyphosate in vetch (N Eyres personal observations). In the medium term, the goal is to maintain diverse crop rotations and use robust herbicide programmes not compromised by resistance, supported by non‐chemical tactics such as harvest weed seed control to progressively exhaust the weed seed bank. These tactics will need to be complemented by regular monitoring, with resistance testing conducted periodically to detect shifts early and guide timely adjustments in weed management strategies.

## CONFLICT OF INTEREST STATEMENT

The authors have no conflicts of interest to declare.

## Supporting information


**Table S1.** ALS‐inhibiting herbicides used in preliminary resistance screening.
**Table S2**. Preliminary screening of *Bromus diandrus* populations with ALS‐inhibiting herbicides. Populations were treated with the recommended rates of various ALS‐inhibiting herbicides and their survival expressed as a percentage of the total number of plants treated. Survival of 0% indicates that all plants were killed, whilst 100% indicates that all plants survived and produced healthy new growth. Intermediate survival values indicate that a certain proportion of plants survived and grew, and the rest were killed.
**Fig. S1**. Collection site of *Bromus* populations sampled from the Western Australian grain belt in late 2023. The red pin denotes the location where the two populations (6–24 and 7–24) with resistance to imidazolinone herbicides were collected prior to crop harvest.
**Fig. S2**. Phenotypes of *Bromus diandrus* plants scored as dead (top panel) *vs*. surviving (bottom panel) at 28 days post‐treatment with ALS‐inhibiting herbicides.
**Fig. S3**. Dose–response curves for target‐site resistant populations 6–24 and 7–24 (pooled data), one metabolic‐resistant population 178–21 and one susceptible control population 1–23 treated with a range of doses of (A) sulfometuron or (B) imazamox+imazapyr (co‐formulated imazamox 33 g L^−1^ + imazapyr 15 g L^−1^). Curves were fitted using the drc package in R.
**Fig. S4**. Representative pyrograms showing sequences obtained at positions Pro‐197, Ser‐653 and Trp‐574 of the *Bromus diandrus ALS* gene. All sequenced individuals from all populations had wildtype codons at Pro‐197 (A) and Ser‐653 (B). Susceptible populations 1–23 and 178–21 had wildtype codons at Trp‐574 (C), whilst resistant populations 6–24 and 7–24 had both wildtype and mutant codons at Trp‐574 (D).

## Data Availability

The data that support the findings of this study are available from the corresponding author upon reasonable request.
