# Supplementary material for: A Trp574–Leu substitution confers resistance to three classes of acetolactate synthase inhibitors in Australian brome grass ( B romus diandrus)
Source: Pest Manag Sci. 2026 May 31;82(10):9437–43. doi: 10.1002/ps.70979 (PMC13569260; doi:10.1002/ps.70979)
Supplement: Supplementary file 1 [file PS-82-9437-s001.docx]

**A Trp-574-Leu substitution confers resistance to three classes of acetolactate synthase inhibitors in Australian brome grass (*Bromus diandrus*)**

Roberto Busi^1,2*^, Danica Goggin^1^, Nick Eyres^3^, Fabian Runge^4^, Aimone Porri^5^, and Ken Flower^1^

^1^ Australian Herbicide Resistance Initiative, UWA School of Agriculture and Environment, University of Western Australia, Perth, WA 6009, Australia

^2*^ Current address: Department of Primary Industry and Regional Development Western Australia, 1 Nash Street, Perth, WA 6000, Australia

^3^ Greenough Specialty Agricultural, Greenough, WA 6532, Australia

^4^IDENTXX GmbH, Maybachstrasse 50, 70469 Stuttgart, Germany

^5^BASF SE, Herbicides Early Biology - Global Research & Development Agricultural Solutions, Speyerer Strasse 2, 67117 Limburgerhof, Germany

*Author for correspondence. Email: [roberto.busi@uwa.edu.au](mailto:roberto.busi@uwa.edu.au)

**Supporting information**

**Table S1.** ALS-inhibiting herbicides used in preliminary resistance screening.

| **Active ingredient** | **Formulation** | **Adjuvant** | **Dose (g ha^-1^)** |
| --- | --- | --- | --- |
| Sulfometuron | Oust (750 g kg^‑1^ sulfometuron-methyl)^a^ |  | 25 |
| Sulfosulfuron | Monza (750 g kg^-1^ sulfosulfuron)^b^ |  | 50 |
| Pyroxsulam | Crusader (30 g L^-1^ pyroxsulam + 90 g L^-1^ cloquintocet-mexyl)^c^ | 1% BS1000^b^ | 70 |
| Imazamox+imazapyr | Intervix (33 g L^-1^ imazamox + 15 g L^-1^ imazapyr)^d^ | 1% Hasten^e^ | 16.5+7.5 |

^a^Dupont Australia, Sydney, Australia

^b^Nufarm Australia, Laverton North, Australia

^c^Dow AgroSciences Australia Ltd, Sydney, Australia

^d^BASF Australia Ltd, Melbourne, Australia

^e^Vicchem, Melbourne, Australia

**Table S2.** Preliminary screening of *Bromus diandrus* populations with ALS-inhibiting herbicides. Populations were treated with the recommended rates of various ALS-inhibiting herbicides and their survival expressed as a percentage of the total number of plants treated. Survival of 0% indicates that all plants were killed, whilst 100% indicates that all plants survived and produced healthy new growth. Intermediate survival values indicate that a certain proportion of plants survived and grew, and the rest were killed.

|  | Plant survival (%) | | | |
| --- | --- | --- | --- | --- |
| Population | Sulfometuron | Sulfosulfuron | Pyroxsulam | Imazamox+imazapyr |
| 1-23 | 0 | 37.5 | 0 | 0 |
| 178-21 | 6 | 90 | 87.5 | 0 |
| 6-24 | 100 | 100 | 100 | 100 |
| 7-24 | 100 | 100 | 100 | 100 |

**Fig. S1.** Collection site of *Bromus* populations sampled from the Western Australian grain belt in late 2023. The red pin denotes the location where the two populations (6-24 and 7-24) with resistance to imidazolinone herbicides were collected prior to crop harvest.

**Fig. S2.** Phenotypes of *Bromus diandrus* plants scored as dead (top panel) vs. surviving (bottom panel) at 28 d post-treatment with ALS-inhibiting herbicides.

**Fig. S3.** Dose-response curves for target-site resistant populations 6-24 and 7-24 (pooled data), one metabolic-resistant population 178-21 and one susceptible control population 1-23 treated with a range of doses of (A) sulfometuron or (B) imazamox+imazapyr (co-formulated imazamox 33g L^-1^ + imazapyr 15g L^-1^). Curves were fitted using the drc package in R.

**Fig. S4.** Representative pyrograms showing sequences obtained at positions Pro-197, Ser-653 and Trp-574 of the *Bromus diandrus ALS* gene. All sequenced individuals from all populations had wildtype codons at Pro-197 (A) and Ser-653 (B). Susceptible populations 1-23 and 178-21 had wildtype codons at Trp-574 (C), whilst resistant populations 6-24 and 7-24 had both wildtype and mutant codons at Trp-574 (D).
